# Effects of advance care planning in care dependent community-dwelling older persons (STADPLAN): A cluster-randomised controlled trial

**DOI:** 10.1177/02692163231180322

**Published:** 2023-06-13

**Authors:** Falk Hoffmann, Rieke Schnakenberg, Katharina Silies, Almuth Berg, Änne Kirchner, Julia Jaschke, Burkhard Haastert, Birgitt Wiese, Juliane Köberlein-Neu, Gabriele Meyer, Sascha Köpke

**Affiliations:** 1 Department of Health Services Research, School of Medicine and Health Sciences, Carl von Ossietzky University Oldenburg, Oldenburg, Germany; 2 Institute for Social Medicine and Epidemiology, Nursing Research Unit, University of Lübeck, Germany; 3 Medical Faculty, Institute for Health and Nursing Science, Martin Luther University Halle-Wittenberg, Halle (Saale), Germany; 4 Center for Health Economics and Health Services Research, Schumpeter School of Business and Economics, University of Wuppertal, Wuppertal, Germany; 5 mediStatistica, Wuppertal, Germany; 6 Institute for General Practice, Hannover Medical School, Hannover, Germany; 7 Institute of Nursing Sciences, University of Cologne, Medical Faculty and University Hospital Cologne, Cologne, Germany

**Keywords:** Randomised controlled trial, end-of-life care, advance care planning, nurses, home care services

## Abstract

**Background:**

Most randomised controlled trials on advance care planning were conducted in people with advanced, life-limiting illnesses or in institutional settings. There are few studies on its effect in older people living in the community.

**Aim:**

To determine the effects of advance care planning in older community dwelling people.

**Design:**

The STADPLAN study was a cluster-randomised trial with 12 months follow-up. The complex intervention comprised a 2-days training for nurse facilitators that delivered a formal advance care planning counselling and a written information brochure. Patients in the control group received optimised usual care, that is, provision of a short information brochure.

**Setting/participants:**

Home care services in three regions of Germany were randomised using concealed allocation. Care dependent clients of participating home care services, aged 60 years or older, and rated to have a life-expectancy of at least 4 weeks were included. Primary outcome was active participation in care at 12 months, assessed by blinded investigators using the Patient Activation Measure (PAM-13).

**Results:**

Twenty-seven home care services and 380 patients took part. Three hundred seventy-three patients were included in the primary analysis (*n* = 206 in the intervention and *n* = 167 in the control group). There was no statistically significant difference between the intervention and control group with regard to the PAM-13 after 12 months (75.7 vs 78.4; *p* = 0.13). No differences in quality of life, anxiety and depression, advance care planning engagement, and in proportion of participants with advance directives were found between groups.

**Conclusions:**

The intervention showed no relevant effects on patient activation or quality of life in community dwelling older persons, possibly indicating the need for more tailored interventions. However, results are limited by a lack of statistical power.

**Trial registration:**

German Clinical Trials Register: DRKS00016886

**What is already known about the topic?**

- Advance care planning can help to inform health care providers and relatives about a person’s priorities, beliefs, values and choices and, therefore, expanding advance care planning to all older people has been demanded.
- Most studies on the effects of advance care planning in older people were conducted in institutional settings such as hospitals or nursing homes and only few studies from European countries are available.

**What this paper adds**

- Our cluster-randomised controlled trial conducted in 27 home care services with 380 patients showed no influence of a formal advance care planning counselling by nurse facilitators on patients’ active participation in their care after 12 months of follow-up, but our study is limited by a lack of statistical power.
- This complex advance care planning intervention also had no effect on quality of life, anxiety and depression, advance care planning engagement and the proportion of participants with advance directives.

**Implications for practice, theory or policy**

- The intervention primarily focusing on promoting patients’ awareness had no effect on any of our assessed endpoints. Therefore, different endpoints and possibly more tailored interventions should be used in future studies.
- In the light of the more recent literature, there seem to be growing evidence that advance care planning interventions do not alter patient-reported outcomes but seem to improve advance care planning discussion and documentation, which seems specifically important, when palliative care is provided.

## Introduction

Advance care planning (ACP) enables individuals to define and record goals and preferences regarding future medical treatment and care in case of physical or mental deterioration.^1,2^ This communication process takes place between individuals and skilled care providers (facilitators) and may also involve relatives. As ACP actively informs health care providers and relatives on a person’s priorities, beliefs and values, it extends a person’s autonomy to a phase in life where they become incapacitated.^3^

Although there is abundant literature on ACP, few randomised controlled trials on its effects are available. An early and comprehensive systematic review published in 2014 found 113 studies on the effectiveness of ACP, but most were observational studies and only six randomised controlled trials were included.^4^ Since then, the effectiveness of ACP has mostly been studied for people with advanced, life-limiting diseases like patients with cancer or heart failure.^5,6^ However, with ageing of Western populations leading to a further increase in care dependent, chronically ill as well as functionally and cognitively impaired older people,^7,8^ expanding and adopting ACP to all older people is of major importance.^1,9^ Most studies focusing on its effects in older people were conducted in institutional settings such as hospitals or nursing homes, while only few studies were performed in the community,^9^ which is the most important setting where older care dependent people live. Furthermore, almost all randomised controlled trials on the effectiveness of ACP in community dwelling older people were conducted in the United States or Canada.^9–11^ The only study from European countries was undertaken in the Netherlands, but it included both nursing homes residents and community dwelling older people.^12^

Therefore, the aim of this study was to evaluate the effect of an ACP program on patients’ activation regarding healthcare issues in care dependent community dwelling older people in Germany.

## Methods

### Study design

STADPLAN (STudy on ADvance care PLANning in care dependent community dwelling older persons) was a multi-centre, two-arm cluster-randomised controlled, pragmatic trial with 12 months of follow-up. Clusters were defined as home care services and randomisation was carried out on cluster level in order to minimise contamination. Detailed information regarding the study protocol of the trial and the process evaluation can be found elsewhere.^13,14^ Results of the comprehensive process evaluation have already been published.^15^

Ethical approval was obtained from the responsible authority in each centre.

### Study setting and participants

Using publicly available registers, home care services were identified in the catchment areas of the three regions Lübeck (north-eastern Germany), Oldenburg/ Bremen (north-western Germany) and Halle (Saale)/Leipzig (eastern Germany). A total of 346 home care services were invited to participate between April 2019 and December 2019. They were included if they cared for at least 70 patients and were willing to assign at least two nurse facilitators for training and providing the intervention.

To be eligible, patients had to be (i) clients of a participating home care services, (ii) aged 60 years or older, (iii) assigned to a care grade (indicating being care dependent and receiving benefits covered by long-term care insurance^16,17^) and (iv) estimated to have a life-expectancy of at least 4 weeks. Furthermore, (v) adequate German language skills and (vi) the cognitive ability to give informed, follow the intervention and participate in data collection were required. Eligibility criteria were assessed by the home care services.

### Randomisation

Computer-generated lists were used with fixed block sizes of two home care services. Therefore, pairs of clusters were randomised at the same time. After assessing baseline characteristics of patients, clusters were randomly assigned by one investigator (JKN) not involved in the recruitment process ensuring concealed allocation.

### Intervention and control

The development of the complex intervention is described elsewhere.^18^ In brief, our intervention was designed in accordance with the Medical Research Council’s (MRC) framework,^19^ based on the Respecting Choices® program,^20^ an established ACP approach, which follows the ethical principles of informed consent, best interest and shared decision making.^21^ The intervention and its implementation were conducted on the levels of (i) the home care services and (ii) the patient (Supplemental eTable 1) with the main aim of raising awareness for ACP on both levels rather than supporting completion of advance directives.

On the cluster level, nurse facilitators employed by the respective home care services received a 2-days training to be prepared for their role as facilitator. This workshop provided an introduction on and the possibilities of documenting ACP. Nurse facilitators were trained to lead conversations based on standardised topic guides developed in the STADPLAN study. Conversation experiences and problems, as well as coping strategies were discussed. To participate, nurse facilitators had to have a minimum qualification of 3-year vocational training.

On the patient level, the intervention included a formal ACP counselling including two conversations (being at least 2 weeks apart) and a written information brochure, both provided by the nurse facilitators. The brochure supported the discussion of health care preferences and wishes regarding future treatment by reflective questions. It further included a glossary of medical and legal terms as well as contact information on local consultancies. In the first conversation (about 30 min), the brochure was delivered and explained. During the second conversation (about 60 min) nurse facilitators discussed questions regarding the brochure as well as attitudes, preferences and values of the patients regarding future medical treatment and care. Conversations followed semi-structured topic guides and took place at the patient’s home. The patient’s proxy decision-maker or another person of trust, if available, was also invited to take part.

All intervention components were piloted in four home care services including conversations with 36 patients in order to test feasibility and acceptability.^13^

Patients in the control group received optimised usual care, that is, provision of a short information brochure on ACP. Apart from the interventions, intervention and control group clusters were treated equally.

### Outcome measures and data collection

Primary outcome was patients’ active participation in their care at 12 months, assessed by the Patient Activation Measure (PAM-13)^22^ in the German version.^23,24^ It consists of 13 items and is a valid and reliable instrument measuring the degree to which individuals take an active role in managing their own health, the corresponding health care and its consequences, and the extent to which they feel competent to fulfil this role. Raw scores are added up (range 0–100) with higher scores indicating more participation.^23^ Up to four missing items were imputed by the mean of the other items.

Secondary outcomes were self-reported health related quality of life (VR-12 with 12 items),^25,26^ anxiety and depression (HADS-A and HADS-D with 14 items),^27^ ACP engagement (4 items) on readiness to pass the process^28^ and the proportion of participants with ACP documents. Further secondary endpoints on health services use were assessed in the health economic evaluation^13^ that will be published later.

Data measurement was conducted at baseline (t0), after 6 months (t1) and after 12 months (t2). Most of these data were assessed in face-to-face interviews with patients at their homes. The PAM-13 was assessed via face-to-face or telephone interviews by trained study assistants who were blinded to group allocation of clusters. Data routinely documented were collected during visits at the home care service (e.g. patients’ long-term care grades,^16^ comorbidities, health services used).

### Sample size calculation

We aimed to detect an effect size of 0.35 (Cohens *d*) in the PAM-13 score after 12 months between the intervention and the control group with 90% power (β = 0.10) using a two-sided significance level of 5% (α = 0.05). Based on the *t*-test, a non-cluster study would require a total of 173 patients per group. Assuming an intra-cluster correlation coefficient (ICC) of 0.05^29^ and an average cluster size of 30 participants, 15 clusters would have been required per group. Accounting for the possibility of loss to follow-up, we planned to include 32 clusters with a total of 960 participants.

### Statistical analysis

Analyses were conducted by a statistician (BH) blinded to group allocation of clusters. All analyses were cluster-adjusted following the intention-to-treat principle.

Baseline characteristics were displayed descriptively. The primary outcome (PAM-13) was compared between intervention and control group using a linear mixed model and adjusted for baseline value (fixed effect) and random (cluster) effects. An additional baseline adjusted linear mixed model was fitted to investigate the course of the primary outcome in both time points t1 and t2. This model includes time and interaction time × group as additional fixed effects and was further adjusted for repeated measurement (covariance pattern structure general). For patients, who terminated the study early, imputation was performed using last observation carried forward (LOCF). Sensitivity analyses were conducted as complete case analysis without last observation carried forward. The same analytic approach was used for secondary endpoints. The binary secondary outcomes were aggregated as percentages on cluster level, and ordinary linear models were fitted. On cluster level these models are by itself cluster adjusted and differences of percentages can easily be estimated from the model.

Statistical tests were performed two-sided at a significance level of α = 0.05 and 95% CI were estimated.

All statistical analyses were conducted with SAS for Windows version 9.4 (SAS Institute Inc, Cary, North Carolina).

## Results

**Figure 1.**
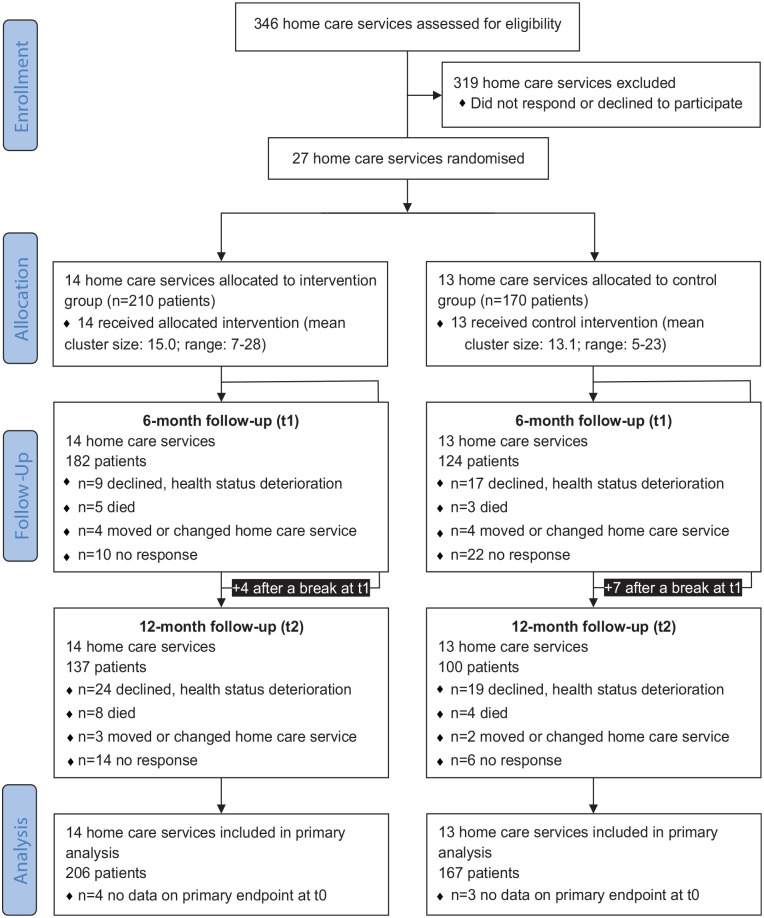
Flow diagram.

Overall, 27 home care services took part (intervention group *n* = 14; control group *n* = 13, see Supplemental eTable 2 for characteristics of home care services). Overall, 380 patients were included (intervention group *n* = 210; control group *n* = 170, see Figure 1). All clusters completed the study. Data on the primary outcome were available for *n* = 373 patients at baseline, *n* = 282 after 6 months and *n* = 233 after 12 months of follow-up. A total of *n* = 373 patients were included in the primary analysis. Of the 210 patients randomised to the intervention group, 184 received the first and 147 also the second conversation.

Baseline characteristics of included patients show that groups were well-balanced (Table 1). Participants’ mean age was 80 years and about two thirds were female. One third had been hospitalised at least once during the last 3 months and more than 80% had at least substantial limitations in activities of daily living (care grade ⩾2).

**Table 1.** Baseline characteristics of patients (*n* = 380).

Characteristics^a^	Intervention group (*n* = 210)	Control group (*n* = 170)
Age in years (*n* = 376)		
Mean (SD)	79.7 (8.9)	80.2 (8.6)
Median [IQR]	81 [75–86]	82 [75–86]
Female sex (*n* = 379)	137 (65.6%)	117 (68.8%)
Living alone (*n* = 371)	119 (58.6%)	110 (65.5%)
Long-term care grade^b^ (*n* = 370)		
None/1 (no or minimal care need)	29 (14.1%)	28 (17.0%)
2 (substantial care need)	116 (56.6%)	107 (64.8%)
3 (severe need)	50 (24.4%)	28 (17.0%)
4/5 (very severe need)	10 (4.9%)	2 (1.2%)
Comorbid diagnoses, lifetime prevalence		
Heart diseases (*n* = 375)	119 (57.2%)	88 (52.7%)
Fractures (*n* = 377)	97 (46.6%)	86 (50.9%)
Diabetes (*n* = 377)	63 (30.0%)	57 (34.1%)
Cancer (*n* = 379)	55 (26.2%)	55 (32.5%)
Stroke (*n* = 370)	41 (19.5%)	45 (26.9%)
COPD (*n* = 378)	29 (13.8%)	16 (9.5%)
Parkinson’s disease (*n* = 378)	10 (4.8%)	9 (5.3%)
Dementia (*n* = 371)	7 (3.4%)	9 (5.5%)
Hospitalisation, last 3 months (*n* = 373)	60 (29.3%)	61 (36.3%)

COPD, chronic obstructive pulmonary disease.

a Number of participants differs from *n* = 380 because of missing values.

b Care grades are assigned upon application and evaluation. Recipients receive benefits covered by the long-term care insurance depending on the assessed care need. The five different care grades focus on the individual ability to manage sustained physical, cognitive, or psychological impairments or health-related stresses or requirements.^16,17^

The intra-cluster correlation coefficient for the PAM-13 at baseline was 0.0873 versus 0.1570 in the intervention and control group, respectively (Supplemental eTable 3). When adjusted for baseline, the primary outcome (PAM-13) did not differ significantly between the intervention and control group (75.7 vs 78.4; *p* = 0.13) after 12 months of follow-up (Table 2). The same result was found in the complete case analysis. We also did not find differences in the PAM-13 between the intervention and control group for the 6-months follow-up as well as significant changes within groups between 6 and 12 months (Supplemental eTable 4).

**Table 2.** Primary and secondary outcomes.

Outcome	Baseline (*t*0)^a^		12-Month follow-up (*t*2)^b^		Difference (95% CI)	*p* Value
	Intervention group (95% CI)	Control group (95% CI)	Intervention group (95% CI)	Control group (95% CI)		
PAM-13 (*n* = 373)	77.9 (74.3–81.5)	79.0 (74.7–83.3)	75.7 (73.2–78.2)	78.4 (75.8–81.1)	2.7 (−0.9 to 6.4)	0.1313
HADS-A (*n* = 368)	5.1 (4.4–5.8)	5.5 (4.5–6.5)	4.9 (4.5–5.4)	4.9 (4.4–5.3)	−0.1 (−0.7 to 0.6)	0.8139
HADS-D (*n* = 368)	6.0 (5.4–6.6)	6.8 (6.1–7.5)	6.5 (6.1–6.9)	6.3 (5.9–6.8)	−0.2 (−0.8 to 0.4)	0.4718
VR12-PCS (*n* = 375)	25.5 (24.0–27.1)	24.2 (21.3–27.1)	24.6 (23.2–26.0)	25.5 (24.0–27.0)	0.9 (−1.1 to 3.0)	0.3637
VR12-MCS (*n* = 374)	47.6 (45.6–49.6)	47.0 (43.8–50.3)	47.7 (45.9–49.5)	49.0 (47.1–51.0)	1.3 (−1.3 to 3.9)	0.3230
ACP-Engagement-4 (*n* = 349)	3.7 (3.5–3.9)	3.9 (3.7–4.1)	3.9 (3.8–4.1)	3.8 (3.7–4.0)	−0.1 (−0.3 to 0.1)	0.3504
Power of attorney^c^ (%) (*n* = 27 cluster, 376 patients)^c^	72.5 (66.3–78.6)	72.7 (63.1–82.3)	74.9 (69.8–80.1)	76.3 (70.9–81.7)	1.4 (−6.1 to 8.8)	0.7052
Appointment of legal representative^c^ (%) (*n* = 27 cluster, 355 patients) ^c^	29.5 (16.7–42.2)	22.7 (14.5–30.8)	37.4 (27.1–47.6)	41.2 (30.5–51.8)	3.8 (−11.2 to 18.7)	0.6067
Advance directive^d^ (%) (*n* = 27 cluster, 371 patients) ^e^	63.4 (55.0–71.7)	63.8 (54.9–72.8)	71.3 (65.5–77.1)	66.7 (60.6–72.8)	−4.6 (−13.0 to 3.8)	0.2699

a Means at baseline from all patients with non-missing values at t0 and LOCF values at t2; 95% CIs cluster adjusted.

b Model-based and adjusted for baseline (mean value of both groups at *t*0 assumed fixed effect at baseline) with last observation carried forward (LOCF).

c Power of attorney and appointment of representatives authorise surrogate decision making on defined areas.

d Advance directives contain treatment preferences in case of health deterioration.

e Mean percentages on cluster level and 95% CIs on cluster level, linear model on cluster level.

For all secondary outcomes, no statistically significant differences were found. Neither the VR-12 (VR-12-PCS: 24.6 vs 25.5, *p* = 0.36; VR-12-MCS: 47.7 vs 49.0, *p* = 0.32) nor the HADS (HADS-A: 4.9 vs 4.9, *p* = 0.81; HADS-D: 6.5 vs 6.3, *p* = 0.47) differed between the intervention and control group after 12 months. There were also no differences between groups in the proportion of patients having a power of attorney, an appointment of legal representative or an advance directive (Table 2). Results did not differ in the complete case analysis (Supplemental eTable 5). When assessed on an individual level for those with complete data for t0 and t2, the proportion of patients having ACP documents increased in both groups. For instance, at baseline 26.4 versus 20.7% of patients in the intervention versus control group held an appointment of legal representative, which increased to 44.2 versus 50.0% after 12 months (*n* = 221, see Table 3).

**Table 3.** Change of proportion of patients with advance care planning documents (complete case analysis, individual level).

Outcome	Baseline (*t*0)		12-month follow-up (*t*2)	
	Intervention group (95% CI)	Control group (95% CI)	Intervention group (95% CI)	Control group (95% CI)
Power of attorney (%) (*n* = 234)	69.9 (62.0–77.7)	73.5 (61.5–85.4)	72.8 (65.2–80.4)	79.6 (70.9–88.3)
Appointment of legal representative (%) (*n* = 221)	26.4 (11.7–41.0)	20.7 (11.4–29.9)	44.2 (33.4–55.0)	50.0 (31.0–69.0)
Advance directive (%) (*n* = 235)	62.5 (52.5–72.5)	68.7 (57.7–79.6)	77.2 (68.3–86.1)	73.7 (64.9–82.6)

Percentages on individual level and cluster adjusted CI.

## Discussion

For our cluster-randomised controlled trial, we were only able to recruit about 40% of the planned sample size, limiting the study’s statistical power, although the planned number of home care services was almost reached. The ACP intervention provided by nurses of home care services did neither significantly affect patient activation nor quality of life after 12 months. In both groups the proportion with appointments of surrogates increased with no significant differences between groups.

A Dutch study recently published by Overbeek et al. also used the PAM-13 as primary endpoint and did not find significant effects of a nurse-led ACP intervention in nursing home and home care settings after 12 months.^12^ The same was true for quality of life and patient satisfaction, which is comparable to our results. A recent systematic review with meta-analysis including 21 randomised controlled trials on different non-physician led palliative care interventions including ACP in patients with advanced, life-limiting illness also did not find effects on quality of life, anxiety and depression, both shortly after the intervention and later.^6^ No effect on quality of life was also found in the large multinational European ACTION trial conducted in more than 1100 patients with advanced cancer.^30^ Earlier studies often did not assess these outcomes.^31,32^ Although this might be striking, our study provides further evidence that ACP does not seem to affect important patient-reported outcomes like patient activation, quality of life, depression and anxiety. The body of evidence is somewhat different for effects on completion of advance directives or appointing surrogate decision-makers, for which we did not find a statistically significant difference between both groups. The Dutch study found large differences regarding this outcome.^12^ The same is true for a recently published randomised controlled trial in older multimorbid outpatients conducted in primary care practices in North Carolina^10^ and for the meta-analysis of palliative care interventions in patients with advanced, life-limiting illnesses^6^ as well as for a recent scoping review on randomised controlled trials on ACP in adults.^32^ However, one has to keep in mind that most interventions evaluated also include the completion of advance directives,^6,9,31^ which was explicitly not part of our program. Although providing advance directives obviously results in more persons completing them, successful implementation of ACP requires training of health care staff and raising awareness of all persons involved.^33^ Therefore, the STADPLAN intervention solely focused on raising awareness and activating participants by providing information and counselling.^13^ The implementation strategy supports comparability of the intervention, but also enables home care services to participate despite scarce staff resources and tight financial margins. Recruitment difficulties confirmed this assumption. Still, the process evaluation showed good knowledge, self-perceived competence and motivation of nurses.^15^

In our study, on the other hand, the completion of appointments of surrogates increased in both groups during follow-up. Other studies lack information on this at baseline.^10,12^ Interestingly, in an older randomised controlled trials by Brown et al. mailing of written materials alone already substantially increased the placement of advance directives, but the provision of a 20-min videotape added no further effect.^34^ This might be interpreted in the way that already delivering a short written brochure could be effective, which was our minimal intervention in the control group. As data collection was performed at patients’ homes and explicitly asking for existing documents, date of signature or surrogate appointments, this may also have increased awareness in patients or their proxy decision-makers. Another explanation could be the Hawthorne effect, as home care services in the control group were aware of being in a study aiming to implement ACP and, furthermore, blinding of home care services was not possible. Therefore, it might be assumed that participation in the study raised awareness. We learned from the process evaluation, that the main motivation to participate was that home care services perceived ACP as important and wanted to improve their care. One cluster in the control group even reported having developed their own concept of ACP during the study, although they had to abandon it due to the COVID-19 pandemic.^15^

Our study highlights important issues on conducting research in patients cared for by home care services. On the one hand, it is feasible to conduct randomised trials in home care services and none of those dropped out during our study. Still, recruitment was difficult. We invited a total of 346 home care services of whom eventually 27 took part. The main reasons for non-participation were time-constraints and lack of qualified personnel.^15^ This underlines that only highly motivated organisations participated, probably resulting in a positive selection. Recruitment of patients was perceived as difficult by home care services. They reported it to be time-consuming as the study topic and the design were difficult for patients to understand. Still, of the 15 home care services documenting their recruitment contacts, a total of 36% of patients participated,^15^ which is exactly the same proportion found in the Dutch study.^12^ Therefore, recruiting home care services seems to be more challenging than recruiting patients. Future research needs to consider tight financial margins and especially lack of nursing personnel as well as that study participation is intrinsically motivated by the topic.

### Strengths and limitations

The main strength of our study was the randomised design with a rigorous method, with outcome assessors blinded to the group allocation for the primary outcome. None of the home care services dropped out during the study.

Our main limitation is that with 380 included patients, only about 40% of the planned sample size of 960 was achieved. Although we were able to recruit 85% of the planned 32 home care services, the number of participating patients was much lower than expected and ranged between 5 and 28 per cluster (with a mean of 14.1 per cluster instead of the planned 30). Furthermore, the number of patients lost to follow-up was comparably high and attrition in the control group was somewhat higher. As we used last observation carried forward imputation for the primary outcome, about 40% of patients’ outcomes at 12 months were imputed with baseline values potentially leading to conservative estimates. However, in the pre-planned sensitivity analyses (complete case analysis and mixed model to investigate the development between t1 and t2), results were comparable. Furthermore, the intra-cluster correlation coefficient was higher than expected, also limiting statistical power of our study. Additionally, generalisability might also be limited as, compared to the general population,^23^ study patients already had high levels of activation and it is possible that some had received some forms of ACP beforehand. There were some deviations from the study protocol.^13^ A few patients were recruited by nurse facilitators that did not fulfil our initial inclusion criteria (e.g., younger than 60 years or having no care grade). It was originally planned to stratify randomisation solely by region, but when pairs were not available, home care services of different regions or a dummy were used. Furthermore, we had to use different methods for data collection. We had planned to assess all endpoints via face-to-face interviews at the patient’s home,^13^ but restrictions due to the COVID-19 pandemic forced us to also allow telephone interviews. However, this was only the case for follow-up contacts (t1 and t2) and the study assistants remained blinded to group allocation. The COVID-19 pandemic might be responsible for the high number of dropouts and it might also have influenced the adoption of central parts of our intervention (e.g., social distancing requirements were occasionally mentioned by home care services to have terminated conversations).

## Conclusion

Overall, we did not find an effect of a concise ACP intervention on levels of patient activation or quality of life in community-dwelling older persons, which contributes to the growing evidence that ACP does not alter patient-reported outcomes. Interestingly, the completion of appointments of surrogates increased in both groups in our study, which might be interpreted in the way that already delivering a short written brochure and introducing ACP could be effective. However, our results are limited by the lack of statistical power and high dropouts. The fact that ACP discussion and documentation may be improved by these interventions, seems important for palliative care provision. Future trials need to identify effective elements of ACP interventions and a further debate on relevant outcomes is needed.

## Supplemental Material

sj-docx-1-pmj-10.1177_02692163231180322 – Supplemental material for Effects of advance care planning in care dependent community-dwelling older persons (STADPLAN): A cluster-randomised controlled trial Click here for additional data file. Supplemental material, sj-docx-1-pmj-10.1177_02692163231180322 for Effects of advance care planning in care dependent community-dwelling older persons (STADPLAN): A cluster-randomised controlled trial by Falk Hoffmann, Rieke Schnakenberg, Katharina Silies, Almuth Berg, Änne Kirchner, Julia Jaschke, Burkhard Haastert, Birgitt Wiese, Juliane Köberlein-Neu, Gabriele Meyer and Sascha Köpke in Palliative Medicine
