# Supplementary material for: Effects of advance care planning in care dependent community-dwelling older persons (STADPLAN): A cluster-randomised controlled trial
Source: Palliat Med. 2023 Jun 13;37(8):1193–201. doi: 10.1177/02692163231180322 (PMC10503242; doi:10.1177/02692163231180322)
Supplement: sj-docx-1-pmj-10.1177_02692163231180322 – Supplemental material for Effects of advance care planning in care dependent community-dwelling older persons (STADPLAN): A cluster-randomised controlled trial [file sj-docx-1-pmj-10.1177_02692163231180322.docx]

**Appendix**

eTable 1. Intervention elements and implementation strategies of the STADPLAN study

| **Intervention group** | **Control group** |
| --- | --- |
| **Home care service level (Implementation strategies)** | |
| **2-day training for participating nurses**  Divided into 7 modules:  M1: Introduction of the STADPLAN study  M2: Introduction of the topic ACP  M3: Practical exercise of the counselling conversations, extensive practice of the conversations based on topic guides with partners using different health situations/cases  M4: Facilitator’s tasks and schedule in the course of the study  M5: Reflexion on conversation experiences  M6: Special practical training of difficult conversational situations, refresher of knowledge on ACP  M7: Feedback and closing of the training | **-** |
| **Structured topic guides for ACP conversations** containing main topics, example prompts and space for noting main results of the conversation |  |
| **Patient level (intervention)** | |
| **ACP counselling (divided into 2 parts)**  **Part 1**: Information on the project, ACP, aim of the conversations, information on the tasks and features of the surrogate/ representative, information on the written living will, introduction of the written information brochure, preparation of the next conversation: topic and goal, presence of a representative  **Part 2**: Repeating information on project, ACP and aim of the conversation, introduction of the following topics: attitudes, preferences and values of the participant, reflection on the use of the additional written information and integration of notes, clarification: further conversations requested? | **-** |
| **Written information on ACP:**  Information brochure of about 60 pages containing:   - Introduction to ACP, surrogate decision making and advance directive documents - Presentation of critical health scenarios along with incapacity - Glossary of medical and legal terms, contact information on local consultancies | **Written information on ACP:**  Brochure of about 15 pages containing:   - Introduction to ACP, surrogate decision making and advance directive documents (condensed) - Presentation of critical health scenarios along with incapacity (condensed) - Glossary of medical and legal terms, contact information on local consultancies |

ACP, advance care planning

eTable 2. Baseline characteristics of home care services (n=27)

| **Characteristics*** | **Intervention group (n=14)** | **Control group (n=13)** |
| --- | --- | --- |
| **Location (n=27)** |  |  |
| Lübeck | 7 (50.0%) | 5 (38.5%) |
| Oldenburg/ Bremen | 4 (28.6%) | 5 (38.5%) |
| Halle (Saale)/ Leipzig | 3 (21.4%) | 3 (23.1%) |
| **Organising institution (n=27)** |  |  |
| Private | 8 (57.1%) | 6 (46.2%) |
| Non-profit | 6 (42.9%) | 6 (46.2%) |
| Public | 0 (0%) | 1 (7.7%) |
| **Total number of patients (n=10)** |  |  |
| Mean (SD) | 233.3 (205.8) | 119.1 (77.9) |
| Median [Min-Max] | 176.5 [68-717] | 133 [17-244] |
| **Number of employees (n=21)** |  |  |
| Mean (SD) | 62.9 (51.0) | 38.4 (20.9) |
| Median [Min-Max] | 48 [24-210] | 27.5 [17-68] |
| **Cooperation with specialised outpatient palliative care service (SAPV) (n=21)** | 8 (61.5%) | 5 (62.5%) |
| **Estimated proportion of patients with advance directives (n=19)** |  |  |
| 0-25% | 6 (54.5%) | 6 (75.0%) |
| 26-50% | 3 (27.3%) | 2 (25.0%) |
| >50% | 2 (18.2%) | 0 (0%) |

* Number of home care services differs because of missing values

eTable 3. Intra-class correlation coefficient (ICC) for primary and secondary outcomes

| **Outcome** | **Intervention group** | **Control**  **group** |
| --- | --- | --- |
| **PAM-13, t0 (n=373)** | 0.0873 | 0.1570 |
| **PAM-13, t2 (n=232)** | 0.0538 | 0.0929 |
| **HADS-A, t0 (n=368)** | 0.0368 | 0.0887 |
| **HADS-A, t2 (n=230)** | 0.0010 | 0.1255 |
| **HADS-D, t0 (n=368)** | 0.0000 | 0.0022 |
| **HADS-D, t2 (n=230)** | 0.0075 | 0.1880 |
| **VR12-PCS, t0 (n=375)** | 0.0202 | 0.1772 |
| **VR12-PCS, t2 (n=233)** | 0.0709 | 0.0523 |
| **VR12-MCS, t0 (n=374)** | 0.0155 | 0.0932 |
| **VR12-MCS, t2 (n=232)** | 0.0328 | 0.2002 |
| **ACP-Engagement-4, t0 (n=349)** | 0.0298 | 0.0080 |
| **ACP-Engagement-4, t2 (n=213)** | 0.0225 | 0.0000 |

t0=baseline; t2=12-month follow-up and ICC derived from the complete case analysis

eTable 4. Development of the PAM-13 throughout the study

|  | **Intervention group**  **(95% CI)** | **Control group**  **(95% CI)** | **Difference**  **(95% CI)** |
| --- | --- | --- | --- |
| **LOCF analysis (n=373)** |  |  |  |
| **6-month follow-up, t1** | 75.4 (73.3 to 77.5) | 77.6 (75.3 to 79.9) | 2.2 (-0.9 to 5.3), p=0.1526 |
| **12-month follow-up, t2** | 75.7 (73.6 to 77.9) | 78.4 (76.0 to 80.7) | 2.6 (-0.6 to 5.9), p=0.1071 |
| **Difference t2 vs. t1** | 0.4 (-1.4 to 2.1), p=0.6934 | 0.8 (-1.2 to 2.7), p=0.4489 |  |
|  |  |  |  |
| **Complete cases (n=278)** |  |  |  |
| **6-month follow-up, t1** | 77.0 (74.3 to 79.6) | 79.6 (76.5 to 82.6) | 2.6 (-1.4 to 6.7), p=0.2012 |
| **12-month follow-up, t2** | 78.4 (75.7 to 81.1) | 81.5 (78.4 to 84.5) | 3.1 (-1.0 to 7.2), p=0.1298 |
| **Difference t2 vs. t1** | 1.4 (-1.4 to 4.2), p=0.3266 | 1.9 (-1.4 to 5.2), p=0.2562 |  |

Results are shown for an additional baseline adjusted linear mixed model that was fitted to investigate the course of the primary outcome in both time points t1 and t2. This model includes time and interaction time*group as additional fixed effects and was further adjusted for repeated measurement (covariance pattern structure general).

eTable 5. Complete case analyses for the primary and secondary outcomes

|  | **12-month follow-up (t2)^a^** | |  |  |
| --- | --- | --- | --- | --- |
| **Outcome** | **Intervention group (95% CI)** | **Control group**  **(95% CI)** | **Difference**  **(95% CI)** | **p-value** |
| **PAM-13 (n=232)** | 78.7 (75.5 to 81.8) | 81.8 (78.3 to 85.2) | 3.1 (-1.6 to 7.8) | 0.1847 |
| **HADS-A (n=230)** | 5.0 (4.4 to 5.6) | 4.7 (4.0 to 5.4) | -0.3 (-1.2 to 0.7) | 0.5610 |
| **HADS-D (n=230)** | 6.3 (5.7 to 7.0) | 6.1 (5.3 to 6.8) | -0.3 (-1.2 to 0.7) | 0.5473 |
| **VR12-PCS (n=233)** | 24.3 (22.4 to 26.2) | 25.7 (23.6 to 27.7) | 1.3 (-1.4 to 4.1) | 0.3297 |
| **VR12-MCS (n=232)** | 48.3 (45.4 to 51.3) | 49.9 (46.7 to 53.1) | 1.6 (-2.7 to 5.9) | 0.4583 |
| **ACP-Engagement-4 (n=213)** | 4.1 (3.9 to 4.3) | 4.0 (3.8 to 4.3) | -0.1 (-0.4 to 0.2) | 0.4829 |
|  |  |  |  |  |
| **Power of attorney (%) (n=27 cluster, 234 patients)^b^** | 67.6 (57.7 to 77.6) | 81.0 (70.7 to 91.3) | 13.3 (-1.0 to 27.6) | 0.0668 |
| **Appointment of legal representative (%) (n=27 cluster, 232 patients) ^b^** | 41.5 (26.1 to 56.9) | 51.4 (35.4 to 67.4) | 9.9 (-12.5 to 32.3) | 0.3692 |
| **Advance directive (n=27 cluster, 235 patients) ^b^** | 76.3 (67.8 to 84.9) | 75.6 (66.7 to 84.4) | -0.8 (-13.1 to 11.5) | 0.8994 |

^a^ Model-based and adjusted for baseline

^b^ Mean percentages on cluster level and 95% CIs on cluster level, linear model on cluster level
